# Supplementary material for: Eco-Geography and Phenology Are the Major Drivers of Reproductive Isolation in the Royal Irises, a Species Complex in the Course of Speciation
Source: Plants (Basel). 2022 Nov 29;11(23):3306. doi: 10.3390/plants11233306 (PMC9739335; doi:10.3390/plants11233306)
Supplement: Supplementary file 1 [file plants-11-03306-s001.zip › plants-1991022-supplementary/S2 AUC maxent.pdf]

plants

AUC – maxent + GLM:

	AUC - maxent	AUC – GLM
I. atrofusca	0.922	0.96
I. atropurpurea	0.930	1
I. bismarckiana	0.917	0.94
I. haynei	0.920	0.93
I. hermona	0.917	0.92
I. lortetii	0.904	0.96
I. mariae	0.896	0.97
I. petrana	0.912	0.96
