# Supplementary material for: Eco-Geography and Phenology Are the Major Drivers of Reproductive Isolation in the Royal Irises, a Species Complex in the Course of Speciation
Source: Plants (Basel). 2022 Nov 29;11(23):3306. doi: 10.3390/plants11233306 (PMC9739335; doi:10.3390/plants11233306)
Supplement: Supplementary file 1 [file plants-11-03306-s001.zip › plants-1991022-supplementary/S3MaxEnt_maps.pdf]

plants

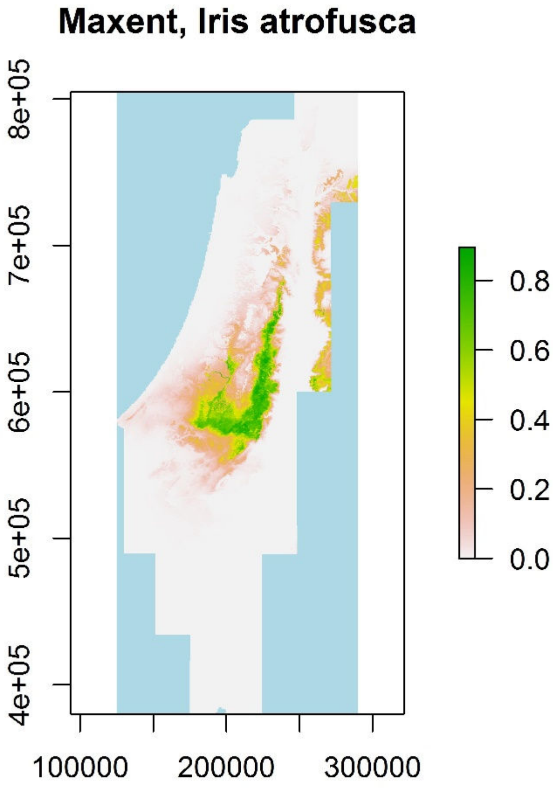
Maxent, Iris atrofusca
8e+05
7e+05
6e+05
5e+05
4e+05
100000
200000
300000
0.8
0.6
0.4
0.2
0.0

A.

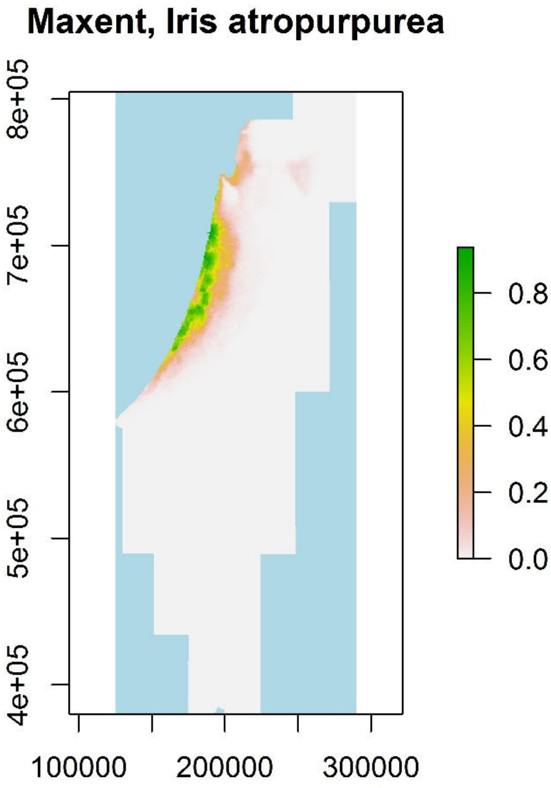
Maxent, Iris atropurpurea
8e+05
7e+05
6e+05
5e+05
4e+05
100000
200000
300000
0.8
0.6
0.4
0.2
0.0

B.

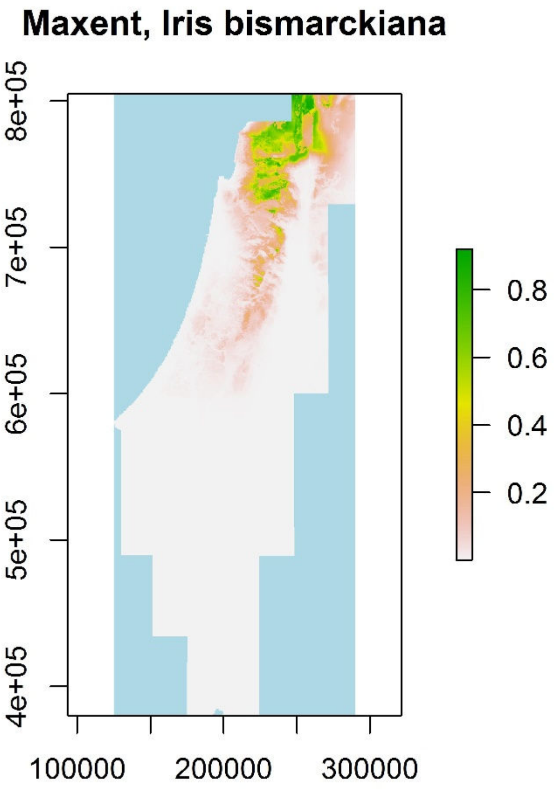
Maxent, Iris bismarckiana
8e+05
7e+05
6e+05
5e+05
4e+05
100000
200000
300000
0.8
0.6
0.4
0.2

C.

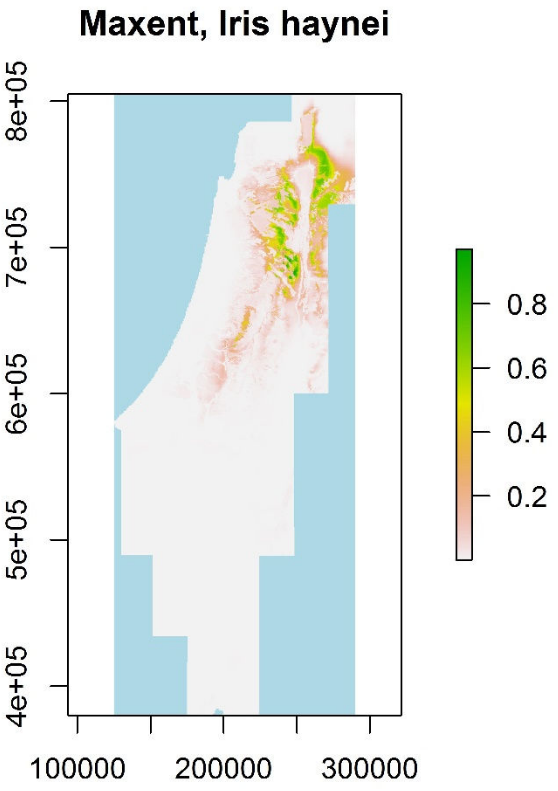
Maxent, Iris haynei
8e+05
7e+05
6e+05
5e+05
4e+05
100000
200000
300000
0.8
0.6
0.4
0.2

D.

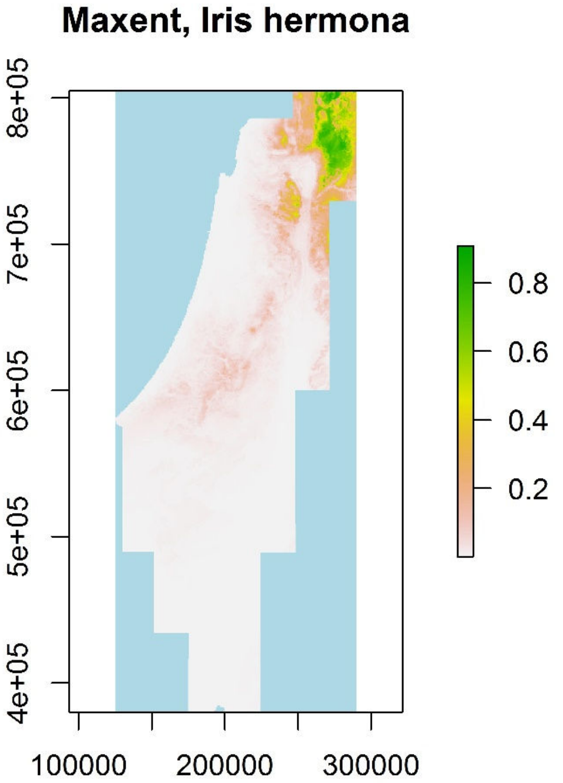
Maxent, Iris hermona
8e+05
7e+05
6e+05
5e+05
4e+05
100000
200000
300000
0.8
0.6
0.4
0.2

E.

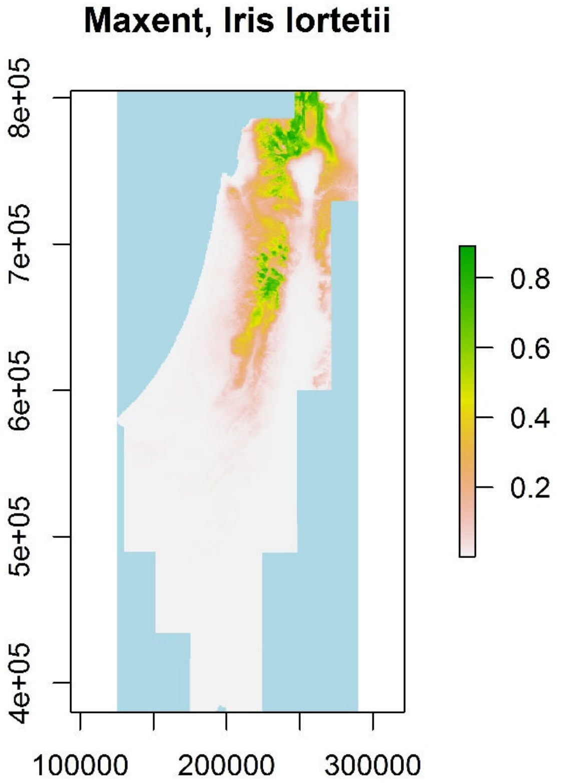
Maxent, Iris lortetii
8e+05
7e+05
6e+05
5e+05
4e+05
100000
200000
300000
0.8
0.6
0.4
0.2

F.

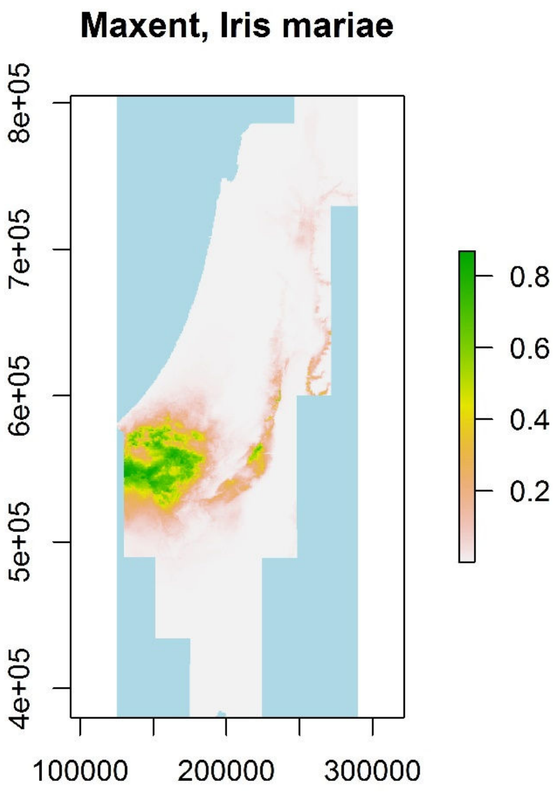

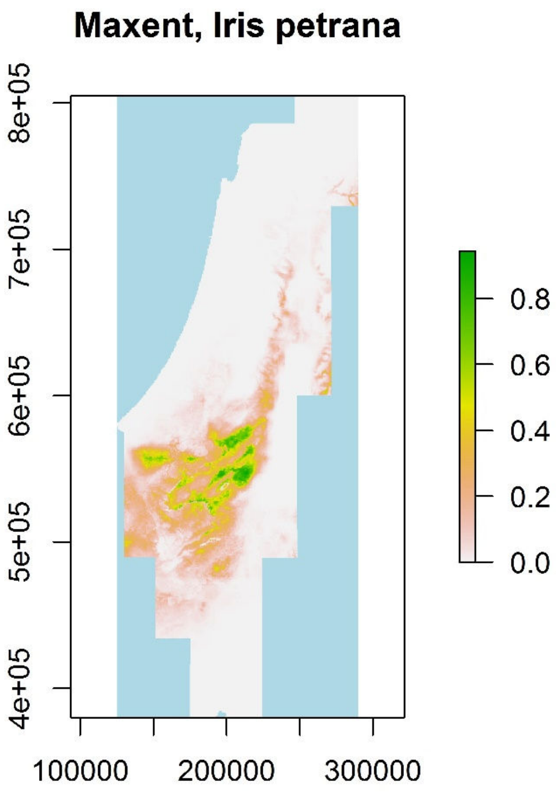

G. H.

Figure S3 – The potential distribution of the eight Israeli *Oncocyclus* Irises, as predicted by the SDMs, performed with MaxEnt. A. *Iris atrofusca*; B. *I. atropurpurea*; C. *I. bismarckiana*; D. *I. haynei*; E. *I. hermona*; F. *I. lortetii*; G. *I. mariae*; H. *I. petrana*.
